# A Singular Case of an Eruptive Disease

**Published:** 1802-08-01

**Authors:** John Badger

**Affiliations:** Little Scotland Yard, Whitehall


### *A singular Case of an Eruptive Disease; communicated by Mr.* John Badger, *of Little Scotland Yard, Whitehall.*

THE firſt opportunity I had of witneſſing this diſeaſe was at Putney, in the month of July, 1801, when it attacked the children in ſeveral families in the neighbourhood, and ſeemed clearly to be confined to children only, to whom it was evidently infectious; indeed, it appears to me to be a diſeaſe peculiar to children of a certain age, having never ſeen a ſingle inſtance of a child being affected with it before 7, nor after 15, though equally expoſed. It commences with a ſlight fever, which continues three or four days; it then increaſes; naufea, and ſometimes vomiting, attend, (in one or two inſtances I have obſerved the patients to complain of violent ſickneſs after they were put to bed) with pain in the head and loins; it is then ſucceeded by an eruption, containing a well maturated pus; the puſtules are large and very thick about the head, reſembling thoſe of the ſmall-pox; and in every caſe which I have ſeen, they have been confined to the head, particularly the ſcalp. (In one inſtance there was a puſtule appeared on the hand, but its contents were very unlike the others, and I much doubt whether it had any affinity to the diſeaſe in queſtion.) The bowels during during the progrefs of the difeafe, were unufually conftipated, and, in one or two inftances, not only the body, but the face likewife was much fwelled. The firft two or three cafes I had not an opportunity of feeing till after the eruption had taken place to a great extent, covering almoft the whole of the fcalp. The gentleman with whom I then refided, whofe medical abilities are well known, confidering it, at firft, only as a common eruption peculiar to children, ordered the hair to be taken off as clofe as poffible, to apply the tar ointment, and to open the bowels with fome mild purgative. This mode of treatment was continued for feveral days without the leaft amendment; the ointment, which was applied every night, feemed rather to increafe than diminifh the number of puftules, and kept them from healing much longer than they otherwife would have been; it was therefore ordered to be omitted, and the head to be kept clean with warm foap and water; the patient to ufe a fpare diet, and the bowels kept open with the pulv. e fcam cum cal. once or twice a week, or pro re nata, and a few drops of antimonial wine given once in four or fix hours, till the feverifh fymptoms had fubfided. This plan was purfued for feveral days without having at all mitigated the complaint, though it feemed, under every circumftance, to be the beft mode of treatment that could be adopted. Accordingly it was continued for a few days longer, at which period the pulfe became regular, the pain in the head and loins was removed, the puftules began to dry off, and in about a week the complaint entirely ceafed.

There were feveral cafes occurred afterwards, and which were cured by the fame treatment.

Since the period abovementioned I have met with feveral fimilar cafes in town, which has given me a greater opportunity of fpeaking more particularly on the fubject, efpecially with refpect to the different modes of treatment, which, under different circumftances, I have found it neceffary to purfue. In the month of May laft, I was requefted to attend a girl about twelve years old; I found her affected with confiderable fever, pulfe quick, tongue white, and a violent pain in her head and loins, accompanied with exceffive thirft, naufea, and coftivenefs; I was informed fhe had been inoculated for the fmall-pox fuccefsfully two years before, otherwife I fhould have pronounced the above fymptoms to have been the forerunner of that difeafe, not at firft confidering the cafe in queftion. I ordered her a mixture with magnes. vitriolat. and mint water, to be given at due intervals, till the bowels were opened freely. The next morning I found her not at all relieved, though the mixture had procured her feveral ftools, but ftill complaining of pain in her head and loins, with an increafe of fever. I ordered her her the faline draughts with vin. antimon. tart. gt. xij. in each, to be taken every four hours, and a low diet. The following day the puftules began to appear on the head, with hard and painful tumours in the glands of the neck; the face fwelled, and likewife the body; fhe had had no ftools but what had been procured her by the opening mixture, which therefore was ordered to be repeated, and to continue the faline draughts every fix hours, with an addition of kali. nitrat. gt. vj. I likewife ordered the head to be fhaved and wafhed night and morning with foap and warm water. This plan I continued for feveral days, when her fever was much abated, and in every other refpect fhe was confiderably better. The puftules difcharged a very thick matter, but feemed very much inflamed round their bafes. I then ordered the following ointment to be ufed night and morning, ℞. Ung. hydrarg. nitrat. cerat. alb. aa. part. æq. and alfo to continue a faline draught every night at bed time. I was happy to find the ointment had the defired effect, for in about a week there was fcarcely the marks of any difeafe remaining, and from that time fhe has continued perfectly well. Two fimilar cafes have occurred to me fince, but having purfued the fame treatment with fuccefs it is needlefs to mention them. However, before I conclude, I think it neceffary to obferve, that though I confider the cafe in queftion as fingular, I do not mean to affert that it has never appeared in print, or to affirm that fimilar inftances have never occurred to any other medical practitioner. Treatifes *may* have been written on the difeafe, which have not come under my obfervation; and though I have not hitherto met with any one who is converfant with it, yet certainly there may be fome few at leaft who are experienced on the fubject; if fuch is the cafe, a communication of their knowledge will be efteemed a favour.

